# Photostability of Isovaline and its Precursor 5-Ethyl-5- methylhydantoin Exposed to Simulated Space Radiations

**DOI:** 10.3390/ijms13011006

**Published:** 2012-01-17

**Authors:** Palash K. Sarker, Jun-ichi Takahashi, Yukinori Kawamoto, Yumiko Obayashi, Takeo Kaneko, Kensei Kobayashi

**Affiliations:** 1 Department of Chemistry and Biotechnology, Faculty of Engineering, Yokohama National University, 79-5 Tokiwadai, Hodogaya-ku, Yokohama 240-8501, Japan; E-Mails: kawamoto-yukinori-tf@ynu.ac.jp (Y.K.); jojo@ynu.ac.jp (Y.O.); t-kaneko@ynu.ac.jp (T.K.); kkensei@ynu.ac.jp (K.K.); 2 NTT Microsystem Integration Laboratories, 3-1 Morinosato Wakamiya, Atsugi 243-0198, Japan; E-Mail: takahashi.junichi@lab.ntt.co.jp

**Keywords:** isovaline, space radiation, ultraviolet light, gamma ray, photolysis

## Abstract

Aqueous solutions of isovaline and its precursor molecule, 5-ethyl-5-methylhydantoin, were irradiated with ultraviolet and γ-ray photons, to evaluate their structural stability against space radiation. The degree of photolysis was measured and irradiation products were identified using chiral, reversed-phase and ion-exchange high-performance liquid chromatography. The experimental results show that the degree of photolysis of 5-ethyl-5-methylhydantoin is more significant than that of isovaline under ultraviolet light irradiation, while the results under γ-ray irradiation are the opposite. As the products of isovaline photolysis, aspartic acid, serine, glutamic acid and alanine were dominantly detected.

## 1. Introduction

A wide variety of bioorganic compounds such as amino acids and their precursors have been detected in carbonaceous chondrites [1,2] and cometary dust [3,4]. Numerous simulation experiments have also suggested their formation pathways from possible astrophysical media by irradiation with high-energy particles [5,6] or ultraviolet (UV) light [7–10]. It is plausible that considerable number of organic compounds, including amino acids and their precursors, were brought to primitive planets by micrometeorites (MMs), comets, and interplanetary dust particles (IDPs) [11]. Among these extraterrestrial bodies, the delivery of MMs and IDPs to Earth at a rate as large as 30,000 tons per year has been observed [12]. Moreover, amino acids in several meteorites (e.g., Murchison, Murray, Orgueil) have been found to have l-enantiomeric excess, the same handedness as observed in some biological amino acids [13,14]. Therefore, it has been suggested that life on Earth was seeded by the delivery of organic compounds from outer space during the intense bombardment period of the primitive Earth [15–17]. For this reason, the photostability of amino acids and their precursors against space radiation could be an important issue in prebiotic chemistry prior to the origin of life on Earth. A number of experiments have already investigated the photostability of free amino acids and their complex precursors against heat, UV light, and cosmic radiation [18–20].

The radiation field in the low Earth orbit (LEO), up to an altitude of 450 km, contains three types of radiation: galactic cosmic radiation (GCR), solar cosmic radiation (SCR), and radiation belts generated by the interaction of trapped GCR and SCR with Earth’s magnetosphere (van Allen belts). GCR, SCR, and the radiation belts are composed of protons, electrons, α-particles, heavy ions, *etc.* [21]. Additionally, the compositions of the solar electromagnetic radiation above the Earth’s atmosphere are 45% infrared radiation, 48% visible light, and only 7% UV light [22]. Although the UV fraction in the solar radiation is small, the photon energies are high enough to excite molecules to electronic excited states and induce photochemical reactions of organic compounds [23].

On the other hand, the polarization of light is an important property with respect to the asymmetric photochemistry of bioorganic molecules. Neutron stars near presolar nebula are the possible astronomical sources of circularly polarized light (CPL) which can trigger enantiomeric excess (EE) of interstellar organic materials through asymmetric photochemical reactions [15,16,24–26]. Recent observations have also shown that massive star-forming regions generate CPL radiation fields spreading over an area as wide as our Solar System [27]. These results suggest that the products with EE through asymmetric photochemical reactions in small bodies of presolar nebula could then have been delivered to the primitive Earth. Several experiments have also examined the asymmetric photochemical reactions of free amino acids in aqueous solution [28–30], and in solid state films [31,32].

**Figure 1:**
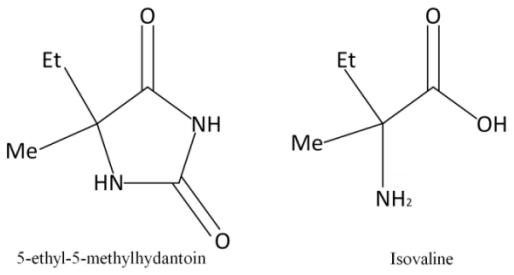
Structure of 5-ethyl-5-methylhydantoin and isovaline.

Isovaline (Ival), a structural isomer of valine and a non-proteinogenic α-methyl amino acid (Figure 1), was found in the Murchison meteorite to display a significant l-enantiomeric excess up to 18.5% due to aqueous alteration of meteorite parent bodies [33,34]. Further, as for one of the precursor molecules of Ival, 5-ethyl-5-methylhydantoin (EM-Hyd), a heterocyclic five-membered ring having α-carbon binding with ethyl and methyl groups (Figure 1), was identified in the Yamato-791198 (24 pmol·g^−1^) and Murchison (47 pmol·g^−1^) meteorites [35]. But their origins and original structure in the chondrites remain controversial, because amino acids and their precursors in meteorites and in experimental simulations of ice irradiations are mostly detected after strong hydrolysis of the residues formed. On the other hand, organic compounds in inner part of comet and meteorites are safe from UV light, but organics in IDPs are fully irradiated with strong solar UV as well as high-energy particles near Earth orbit. So, it is of interest to examine how these organic compounds alter or survive against radiation. In this paper, we report the first phase simulation experiments which have been performed with aqueous solutions of Ival and EM-Hyd against UV and γ-ray irradiation.

## 2. Results and Discussion

### 2.1. Stability of Ival and EM-Hyd Against UV Irradiation

**Figure 2:**
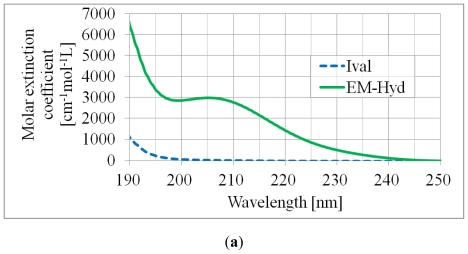

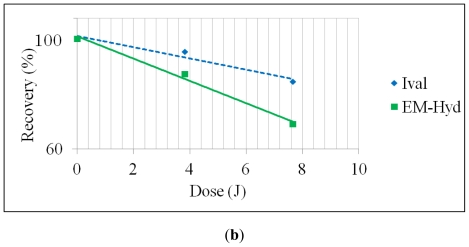
(**a**) UV spectra of Ival and EM-Hyd before irradiation. (**b**) Stability of Ival and EM-Hyd against UV (continuous) irradiation.

The recovery amount (fraction of unphotolyzed molecules in percent) in Table 1 suggests that EM-Hyd was decomposed more significantly than Ival by continuous UV irradiation. Because EM-Hyd has a heterocyclic ring with two carbonyl groups, the strong absorption band appears in the range of 190 to 240 nm, as shown in Figure 2a. The semi-logarithm graph (Figure 2b) also shows the same tendency in the stability difference between Ival and EM-Hyd.

The quantum yield of photolysis, *Φ* (the ratio between the number of photolyzed molecules and the number of absorbed photons), is shown in Table 2. The number of total absorbed photon was calculated according to the Lambert–Beer law (*I*/*I*_0_ = 10^−^*^ɛlc^*, where *I* and *I*_0_ are the intensity of the incident light and transmitted light, respectively, *ɛ* is the molar extinction coefficient, *l* is the length of the solution through which light passes, and *c* is the molar concentration of the solution). As the deuterium lamp (L1835; HAMAMATSU Photonics) produces continuous spectra (190–400 nm), we calculated the average absorbed photon number for every 10 nm of wavelength and obtained the total average absorbed photon number (e.g., ∑*N = N*_190–200_*_nm_+*···*+N*_390–400_*_nm_*, where *N* is the average absorbed photon number). The average *Φ*-value obtained in the continuous UV (190–400 nm) irradiation experiment suggests that approximately 89% of an Ival and 80% of an EM-Hyd molecule can be decomposed by one photon. Although the *Φ* value for UV-photolysis of EM-Hyd is about 9% less than that of Ival, photodecomposition of EM-Hyd is approximately 2~2.5 times larger than that of Ival as EM-Hyd can absorb more UV-photons, which can be clearly understood from Ival’s and EM-Hyd’s molar extinction coefficients, as shown in Figure 2a.

### 2.2. Stability of Ival and EM-Hyd Against γ-Ray Irradiation

**Figure 3:**
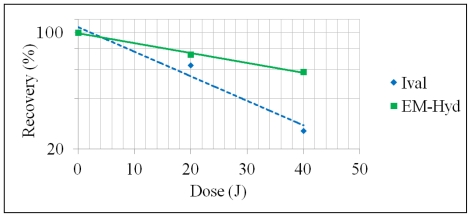
Stability of Ival and EM-Hyd against γ-ray irradiation.

With γ-ray irradiation, Ival was more significantly photodecomposed than EM-Hyd in the same conditions, as shown in Table 1 and Figure 3. The present data strongly imply that Ival can hardly survive under γ-ray irradiation, whereas EM-Hyd undergoes less decay. This result may carry significant implications for the fate of Ival and EM-Hyd under γ-ray irradiation in asteroids and comets; Ival exists probably not in its free form, but rather in a precursor form which may be EM-Hyd.

The quantum yield of photolysis (*Φ*) under γ-ray irradiation for Ival and EM-Hyd is enormously high, as shown in Table 2. This is due to the complex reaction scheme of γ-ray irradiation. A significant number of secondary electrons are generated resulting from the γ-ray interaction with reactant and solvent (H_2_O) molecules (mainly Compton scattering) during irradiation. The secondary electrons and scattered photons then lose energy through a sequence of interactions with the reactant and solvent molecules again. Thus, the amount of decomposition of Ival and EM-Hyd depends on the secondary chain reactions in the radioactive process. As a consequence, the *Φ* value during γ-ray irradiation is four orders of magnitude higher than that during UV irradiation. Because the photon-energy ratio between γ-rays (1.25 MeV) and UV (for example, 6.2 eV at 200-nm wavelength) is 2.0 × 10^5^, the *Φ* value ratio between γ-ray and UV irradiation in this study (Table 2) is reasonable considering the energy loss due to the chain-branching energy-transferring reactions during γ-ray irradiation.

### 2.3. Photolysis Products of Irradiated Ival and EM-Hyd

**Figure 4:**
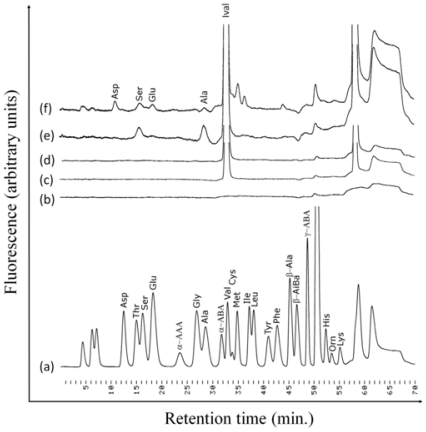
Ion exchange chromatograms of amino acids: (**a**) Wako amino acid standard, (**b**) Blank (milli-Q water), (**c**) Ival without UV irradiation, (**d**) Ival without γ-ray irradiation, (**e**) UV (continuous) irradiated (7.6 J) Ival and (**f**) γ-ray irradiated (40 J) Ival. Abbreviations: Asp: Aspartic acid, Thr: Threonine, Ser: Serine, Glu: Glutamic acid, α-AAA: α-Aminoadipic acid, Gly: Glycine, Ala: Alanine, α-ABA: α-Aminobutyric acid, Val: Valine, Cys: Cystine, Met: Methionine, Ile: Isoleucine, Leu: Leucine, Tyr: Tyrosine, Phe: Phenylalanine, β-ala: β-Alanine, β-AiBa: β-Aminoisobutyric acid, γ-ABA: γ-Aminobutyric acid, His: Histidine, Orn: Ornithine, Lys: Lysine. The IE-HPLC system gives identical retention time for Val and Ival.

**Figure 5:**
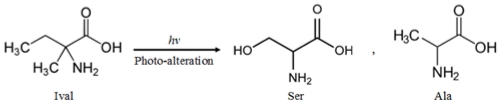
The photo-alteration process of Ival by the energetic photons forms dominantly Ser and Ala.

As the common photolysis products of Ival during continuous UV and γ-ray irradiation, serine (Ser) and alanine (Ala) were formed (Figure 4). The photolysis products were not derived from the contamination, because the amount of photolysis products drastically increased with increasing irradiation dose. Moreover, control experiments were performed and they were compared with actual irradiation experiments in order to verify the photolysis products of Ival. The chromatograms for control samples (*i.e.*, trace b, c and d in Figure 4 for blank milli-Q water, Ival without UV irradiation and Ival without γ-ray irradiation respectively) assured that Ser and Ala were formed as photo-products of Ival. The photo-alteration process by the energetic photons might involve the release of methyl (−CH_3_) and ethyl (−CH_2_CH_3_) groups from Ival in the transition period, which would lead to the formation of Ser and Ala (Figure 5). For the formation of Ser, the OH groups might come from COOH groups, or H_2_O might be another possible source of OH groups as H_2_O could be attacked by newly formed ions during irradiation, although H_2_O molecules do not absorb UV from 190 to 400 nm. Some other amino acids (*i.e.*, aspartic acid, glutamic acid) besides Ser and Ala were formed from Ival by γ-ray irradiation, whereas Ser and Ala were the only common photo-products in the case of UV- and γ-alteration of Ival. This difference might be due to the γ-ray-induced reactions as we mentioned above (see section 2.2). Additionally, two other peaks after the peak of Ival (Figure 4, trace f) could not be identified as their retention times did not match the retention times of the available standards. Further study is required to clarify the precise formation mechanism of the photolysis products generated from irradiated Ival.

The analysis of the photolysis products of irradiated EM-Hyd will be addressed in a future study. As mentioned in the introduction, EM-Hyd is one of the precursors of Ival and it is well known that hydrolysis of EM-Hyd produces Ival [36]. Therefore, it is assumed that EM-Hyd predominantly produces Ival also in the early stage of their photolysis, and finally the same kinds of products are expected as the photolysis of Ival. Other than amino acids, hydantoin, 5-ethylhydantoin, 5,5-dimethylhydantoin, 5-methylhydantoin may be produced as photolysis products from UV and γ-ray irradiated EM-Hyd. Verification study is required to detect the photolysis products of irradiated EM-Hyd.

## 3. Experimental Section

### 3.1. Chemicals

As starting material for the radiation experiment, EM-Hyd (equimolar mixture of d- and l-EM-Hyd) and Ival (equimolar mixture of d- and l-Ival) were abiotically synthesized in the Mita Laboratory at the Fukuoka Institute of Technology, Fukuoka, Japan. Ethylmethylketone was condensed with ammonium carbonate in the presence of potassium cyanide to produce EM-Hyd according to the Strecker synthesis. Then, Ival was synthesized by hydrolyzing EM-Hyd with barium hydroxide. Purity of the synthesized Ival and EM-Hyd was over 99%.

### 3.2. UV Absorption Measurement

UV absorption spectra of Ival and EM-Hyd were measured using a UV-VIS spectrophotometer (JASCO V-660). The wavelength range of the measurement was 190 to 400 nm. Figure 2a shows the absorption spectra only from 190 to 250 nm because no absorption bands of Ival and EM-Hyd were observed from 250 to 400 nm.

### 3.3. UV Irradiation

**Figure 6:**
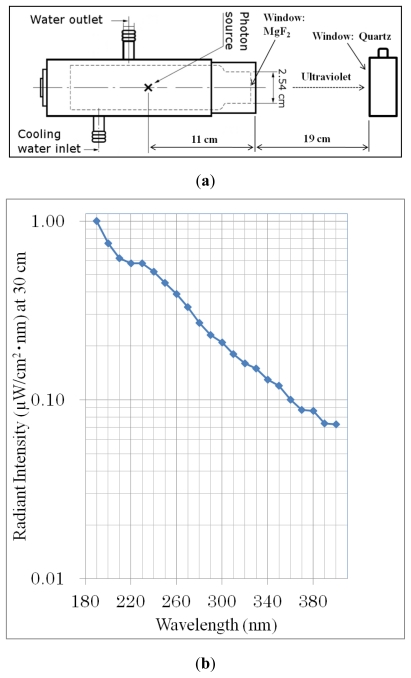
(**a**) Schematic diagram of the experimental setup for UV irradiation (continuous, 190–400 nm) from a deuterium lamp (L1835; HAMAMATSU Photonics). (**b**) Typical spectral irradiance of the deuterium lamp was measured by HAMAMATSU.

Ival and EM-Hyd were placed separately in synthesized quartz cells for UV irradiation with continuous spectra (190 to 400 nm) using a deuterium lamp (L1835; HAMAMATSU Photonics), as shown in Figure 6a. Every sample (2 mL, 10 mM, pH = 7) was irradiated with a power density of 66 μW/cm^2^ for 8 and 16 h at a 30-cm distance from the photon source of the lamp. The total power density emitted from the deuterium lamp was calculated by integrating the spectral irradiance (Figure 6b) over the region 190–400 nm. The beam spot was 6 cm in diameter.

### 3.4. γ-Ray Irradiation

Ival and EM-Hyd were sealed in borosilicate glass tubes in air. Every sample (2 mL, 10 mM, pH = 7) was irradiated with γ-rays from a ^60^Co source (1.25-MeV-photon energy) at the Japan Atomic Energy Agency, Takasaki, Japan. The total irradiation doses were 10 and 20 KGy (20 and 40 J) as absorbed by the solvent.

### 3.5. Analysis of Ival and EM-Hyd

After the UV and γ-ray irradiation, an aliquot of EM-Hyd was hydrolyzed with 6-M HCl at 110 °C for 24 h to convert it into Ival. Following the acid hydrolysis and evaporation to dryness, the hydrolyzed fraction was dissolved in milli-Q water. Finally, a portion of the irradiated sample (Ival, hydrolyzed EM-Hyd) was injected into a chiral chromatographic system to measure the amount of decomposition. The chromatographic system was equipped with a pump (TOSOH DP-8020), a column (Sumichiral OA-5000 or OA-5000L, 4.6 mm i.d. × 150 mm), and a detector (JASCO CD-2095 Plus, 254-nm detection wavelength). Copper (II) sulfate pentahydrate (CuSO_4_·5H_2_O, 1 mM) was used as the mobile phase and the flow rate was 1 mL/min.

In addition, the decomposition of irradiated EM-Hyd was analyzed (without hydrolysis) by reversed-phase high-performance liquid chromatography (RP-HPLC). The RP-HPLC system was composed of two pumps (SHIMADZU LC-20AD), a column (Capcell Pak C_18_ UG120 S-5 μm, 6.0 mm i.d. × 250 mm), and a detector (SHIMADZU SPD-20AV, 210-nm detection wavelength). The temperature of the column was maintained at 37 °C. Phosphate buffer (25 mM, pH = 3.5, flow rate 0.90 mL/min) and acetonitrile (100%, flow rate 0.10 mL/min) were used as the mobile phase under isocratic conditions.

Furthermore, the photolysis products of irradiated Ival were analyzed by ion-exchange high-performance liquid chromatography (IE-HPLC) using post-column derivatization with *o*-phthalaldehyde and *N*-acetyl-l-cysteine. The IE-HPLC system was equipped with two HPLC pumps (Shimadzu LC-10AT), a cation-exchange column (4 mm i.d. × 150 mm; Shimpak ISC-07/S1504), a post column derivatization system, and a Shimadzu RF-535 fluorometric detector (358-nm excitation wavelength; 450-nm emission wavelength). The temperature of the column was maintained at 55 °C. Gradient elution was performed using eluents A (0.07 M trisodium citrate perchloric acid with 7% ethanol, pH 3.20) and B (0.2 M trisodium citrate boric acid with NaOH, pH 10). The flow rate of the carrier was 0.3 mL/min. A Wako amino acid mixture (type AN-II and type B) was used as the amino acid standard for IE-HPLC analysis.

## 4. Conclusions

The present results of laboratory simulation suggest that EM-Hyd (precursor of Ival) is photochemically much more stable under γ-rays and less stable under UV environment than Ival. Moreover, the photo-alteration process of Ival produces Ser and Ala suggesting that Ival and its precursors might play an important role for the formation of Ser and Ala in the prebiotic chemistry. Our second phase simulation experiments will focus on the photochemistry of solid-state samples including glycine (Gly), hydantoin (a precursor of Gly), Ival, EM-Hyd and complex amino acid precursors synthesized from possible interstellar molecules. We are also planning to irradiate them in a real space environment. Recently, the “Tanpopo Mission” [37], a project related to astrobiology, scheduled for June, 2013, has been proposed to catch space dust with ultra-low-density (0.01 g/cm^3^) aerogel and analyze micro-organisms and organic compounds in it. The mission will also involve irradiation of Gly, Ival and their precursors at LEO in a real space environment by using the exposed facility of the Japanese Experimental Module (JEM) on the International Space Station.

## Figures and Tables

**Table 1:** Recovery of Ival and EM-Hyd after UV and γ-ray irradiation. Plus minus (±) represents the standard deviation of multiple analyses.

Types of irradiation	Total energy dose (J)	Recovery (%)	

		Ival	EM-Hyd
UV (continuous, 190–400 nm)	3.8	94 ± 0.24	85 ± 0.95
	7.6	82 ± 0.07	67 ± 0.96
γ-rays	20	64 ± 0.02	74 ± 1.5
	40	26 ± 0.44	58 ± 2.3

**Table 2:** Quantum yield values (*Φ*) for photolysis of Ival and EM-Hyd.

Types of irradiation	Quantum yield of photolysis, *Φ* (molecule/photon)	

	Ival	EM-Hyd
UV (continuous, 190–400 nm)	0.89	0.80
γ-rays	4.4 × 10^4^	2.8 × 10^4^
